# Resuscitative Endovascular Balloon Occlusion of the Aorta as an Adjunct in a Patient with Neurogenic Shock

**DOI:** 10.7759/cureus.3375

**Published:** 2018-09-27

**Authors:** Sanjiv Gray, Beatrice Dieudonne

**Affiliations:** 1 Surgery, University of Central Florida, Orlando, USA; 2 Surgery, Harlem Hospital Center, New York, USA

**Keywords:** reboa, trauma, neurogenic shock, spinal cord injury, vasopressors, massive transfusion

## Abstract

Aortic occlusion is used during trauma resuscitation for patients with profound shock and cardiac arrest. Aortic occlusion increases coronary and cerebral perfusion permitting time for interventions in an attempt to salvage moribund patients. Resuscitative endovascular occlusion of the aorta (REBOA) is a less invasive method of aortic occlusion and its indications are being defined, but it is used primarily for noncompressible torso hemorrhage. This case is the first report of the use of REBOA for neurogenic shock in a trauma patient. The patient presented after a motorcycle accident with altered mental status and hypotension. The patient was resuscitated with blood products and REBOA used as an adjunct to maintain cardiac and cerebral perfusion. The patient underwent stabilization of his spinal injuries and is currently undergoing rehabilitation. There were no complications related to the REBOA.

## Introduction

The case report shows the safe utilization of REBOA in a trauma patient with neurogenic shock. There is a brief description of the patient's presentation and the effect of REBOA on his physiology. This is the first report of the use of REBOA for neurogenic shock. This report will foster further research as clinicians try to define the best indications for the technology.

## Case presentation

**Figure 1:**
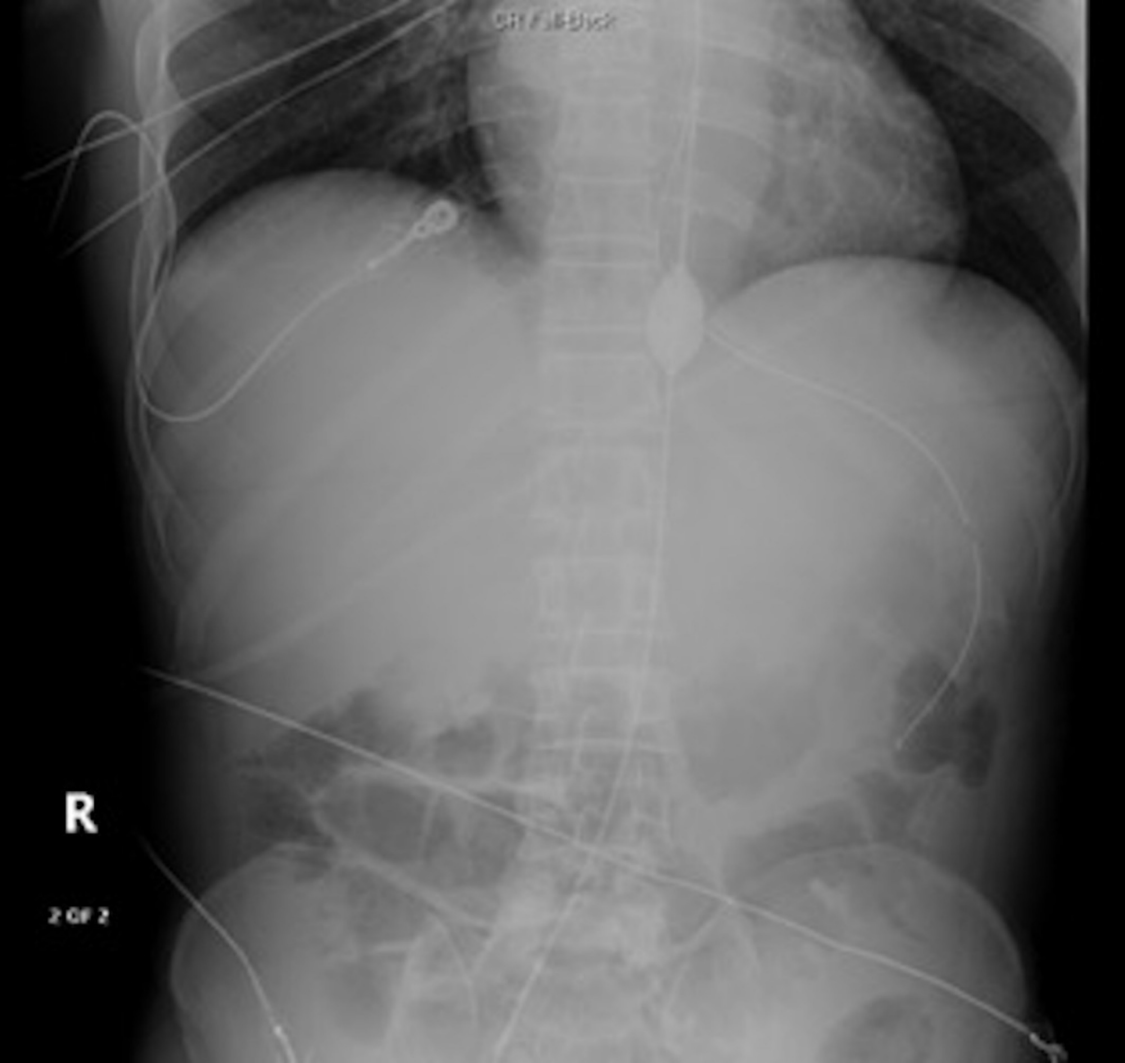
Showing the inflated balloon in aortic zone 1

A 52-year-old male was brought to the trauma center after a high speed motor cycle collision. The patient was not wearing a helmet and had altered mental status on the scene requiring emergent intubation with a King laryngeal tube (Ambu Inc., MD, USA). On arrival at the trauma center his airway was secured with an endotracheal tube and a cervical collar was placed. He had bilateral breath sounds and his oxygen saturation was 100%. His distal pulses were 2+, blood pressure 98/60 mm Hg and pulse rate was 89 beats per minute (bpm). He had a negative focused abdominal examination for trauma (FAST) exam. His Glasgow Coma Score (GCS) was three with pupils two mm bilaterally and reactive. On log roll there was no rectal tone, but the patient had received succinylcholine 100 milligram (mg) for the endotracheal intubation. A foley catheter was placed and no hematuria noted, his pelvis was stable. The patient’s repeat blood pressure was 70/49 mmHg and pulse 85 bpm with a weak thready radial pulse and warm extremities. He was given two units of packed red cells and two units of fresh frozen plasma. His repeat blood pressure was 79/62 mm Hg as measured by a right femoral arterial line. A massive transfusion protocol was initiated with the patient receiving another five units of packed red cells, four units of fresh frozen plasma, one pack of platelets, and one gram of Tranexamic acid. Simultaneously the right femoral arterial line was exchanged for the 7 French introducer catheter and connected to the arterial line with good waveform. The REBOA catheter was placed at 42 centimeter (cm) into zone one. Inflation was done with 2 milliliters (ml), then 4 ml, then 6 ml of saline for a total of 6 ml of saline with improvement in the blood pressure to 112/62 mm Hg. The REBOA catheter was locked and sutured in place. His abdominal X-ray (Figure 1) showed good placement and the catheter was placed to continuous invasive monitoring. His repeat FAST and chest X-ray were negative for intrathoracic effusion or intraabdominal free fluid. Norepinephrine was added for suspected spinal shock given his warm extremities with dilated peripheral veins. Prior to leaving the trauma resuscitation area his systole blood pressure was 125 mm Hg and went up to 195 mm Hg on arrival at the CT scan suite. The REBOA balloon was deflated slowly 30 minutes after inflation and prior to the completion of computed tomography (CT) scans. He was also weaned off the norepinephrine prior to the completion of CT scans, and 0.6 mg of atropine was given for bradycardia in the 40s. He was brought to the intensive care unit for further resuscitation. The patient sustained diffuse axonal injury and subarachnoid hemorrhage. The patient's post resuscitation GCS was 11T. The patient had neurogenic shock secondary to C5 level comminuted fractures involving lateral masses and lamina bilaterally, C6 level comminuted distracted fracture of the spinous process, C7 level fracture of the left lateral mass and left lamina and 5 mm anterolisthesis of C5 on C6 with cord contusion, facet joints causing a perched right C5 facet. The patient also had T1, T2, T3, T5, T7, T8, and T9 level vertebral body fractures and contusion. The patient also had bilateral mandible fracture, avulsion of the right earlobe, and right first and second ribs fracture.

The neurogenic shock resolved and the REBOA catheter was removed 78 minutes after initial placement. The patient did not require further transfusion of blood products or vasopressors. Lovenox for venous thromboembolism prophylaxis was started on hospital day 2. The patient underwent stabilization of his cervical spine and fixation of his mandibular fractures, and reattachment of the right earlobe. At the six-month follow-up the patient was independent of the ventilator, masticated with assistance, and was participating in spinal cord injury rehabilitation.

## Discussion

The optimal use of resuscitative endovascular occlusion of the aorta (REBOA) during trauma resuscitation remains debatable. The main indication for the catheter is for noncompressible torso hemorrhage in patients with profound shock and posttraumatic arrest [1]. More recently other uses for the catheter have been described, such as for postpartum hemorrhage, gastrointestinal hemorrhage, and non-traumatic cardiac arrest [2,3]. Aortic occlusion preserves cerebral perfusion and coronary filling in patient in extremis with refractory hypotension. This leads to increased central aortic pressure, carotid flow, and brain oxygenation [4,5]. The intra-aortic location of the balloon can be divided into three zones for REBOA. Zone 1 extends from the origin of the left subclavian artery to the celiac artery, zone 3 extends from the lowest renal artery to the aortic bifurcation, and zone 2 is the area between where the balloon should not be placed. The length of the aorta differs in each patient and estimated external landmark measurements depend on which catheter is being used. Most placements are done in zone 1 but can be done in zone 3 for patients with hemorrhagic shock secondary to pelvic fractures. Dubose et al. showed improvement in hemodynamics in 62.3% of patients achieved after aortic occlusion with 36% sustaining systolic blood pressure > 90 mm HG for at least five minutes. A Glasgow Coma Score (GSS) of 15 on discharge was noted in 79.2% of survivors [6].

REBOA provides a less invasive method for aortic occlusion especially with the 7 French (Fr) catheters. One can avoid the traditional resuscitative thoracic incision with its associated morbidity in selected cases. There are ongoing studies investigating the optimal approach for aortic occlusion either open or endovascular and there are reports of a hybrid approach utilizing both techniques [7].

Potential complications of REBOA placement includes vascular injuries such as hematoma, pseudoaneurysm, intimal flap, and distal embolization. Utilization of training protocols and skills maintenance are essential to minimize complications while optimizing proper timely placement. The use of the small 7 Fr catheter has been associated with less complications [8]. The use of REBOA in the setting of thoracic injury is potentially dangerous and is considered a contraindication to placement. However minor thoracic injuries such as pulmonary contusion, pneumothorax, and rib fractures are not contraindications to placement. Partial REBOA has been described and used to maintain some distal perfusion and mitigate the effects of prolonged ischemia and reperfusion injury [9,10]. Safe placement and ongoing training are essential as the procedure is being done in the prehospital setting in some trauma systems. In the presented case, REBOA was a crucial adjunct to maintain cerebral and coronary perfusion during the resuscitation and was safely removed without any complications to the patient.

## Conclusions

Although volume replacement and vasopressors are the cornerstone of the management of neurogenic shock, REBOA can be used as an adjunct in carefully selected cases to prevent prolonged hypotension and the risk of anoxic encephalopathy and cardiovascular collapse. Further studies are needed to define the optimal use for REBOA in trauma and different shock states.
